# The efficacy of ultrasound-guided pulsed radiofrequency in the treatment of primary glossopharyngeal neuralgia

**DOI:** 10.3389/fneur.2024.1453598

**Published:** 2024-11-28

**Authors:** Fubo Li, Hongcheng Lu, Gege Gong, Cehua Ou, Yue Zhang

**Affiliations:** ^1^Department of Pain Management, The Affliated Hospital of Southwest Medical University, Luzhou, China; ^2^Department of Physical Diagnosis, The Affliated Hospital of Southwest Medical University, Luzhou, China

**Keywords:** glossopharyngeal neuralgia, pulsed radiofrequency, ultrasound-guided, efficacy, pregabalin, oxcarbazepine

## Abstract

**Objective:**

This study evaluates the clinical efficacy and safety of ultrasound-guided long duration, high voltage pulse radiofrequency (PRF) in managing primary glossopharyngeal neuralgia (GPN).

**Methods:**

Clinical data were retrospectively analyzed for 13 patients with primary GPN who underwent this treatment between August 2019 and October 2022. Visual Analog Scale (VAS) scores were assessed pre-treatment and at 1 week, 1 month, 3 months, and 6 months post-treatment. Additionally, the rates of discontinuation of oral oxcarbazepine and pregabalin, efficacy, and complication rates at 6 months post-procedure were monitored.

**Results:**

Significant post-treatment pain relief, was observed across all patients, with statistically significant improvements in VAS scores (*p* < 0.05). Discontinuation rates for oxcarbazepine and pregabalin were also high (*p* < 0.05). At the 6-month follow-up, 69.23% of patients achieved excellent and good efficacy, 84.61% demonstrated overall effectiveness, while 15.38% showed poor efficacy. No critical complications were reported in any case.

**Conclusion:**

Ultrasound-guided, long-term, high-voltage PRF effectively relieves primary glossopharyngeal neuralgia and improves quality of life. Featuring ease of operation, high safety and minimal complications making it a promising approach for clinical application.

## 1 Introduction

Glossopharyngeal neuralgia (GPN) is a rare condition characterized by sudden, intense, transient, and recurring neuropathic pain in areas including the tongue, throat, and tonsillar fossa. This pain is triggered by specific points in the affected region, with episodes lasting from a few seconds to several minutes. Accompanying symptoms may include bradycardia, hypotension, syncope, convulsions, or even cardiac arrest, posing severe risks to patients’ quality of life and, in some cases, life-threatening outcomes (1, 2). Primary treatment for GPN generally involves first-line medications like carbamazepine and oxcarbazepine, which are well-tolerated but show a high rate of recurrence (3). Prolonged use, however, often leads to diminished efficacy and intolerable side effects, prompting many patients to explore surgical or minimally invasive options. Primary surgical treatments, such as microvascular decompression (4) and glossopharyngeal/vagus nerve sectioning (5), are associated with considerable adverse effects, including hoarseness, dysphagia, and choking, which restrict their clinical applicability (1). For patients unable to undergo or unwilling to consider craniotomy, alternative treatments like extracranial puncture techniques and stereotactic radiosurgery (6) offer similar efficacy with a comparable risk of complications.

Minimally invasive interventions include glossopharyngeal nerve block (7) and percutaneous radiofrequency thermocoagulation (8). The nerve block serves as a diagnostic approach involving local anesthetic application. If effective, additional blocks or percutaneous radiofrequency thermocoagulation of the nerve may be considered. Recently, percutaneous radiofrequency thermocoagulation has shown promising outcomes, with a short-term pain relief rate of 92% (8). However, the procedure may result in non-selective nerve fiber destruction, potentially causing complications such as pharyngeal numbness, vocal cord paralysis, dysphagia, and inadvertent injury to adjacent nerves and blood vessels. Long-term pain relief remains at approximately 50%, limiting its recommendation to specific cases (9, 10).

Pulsed radiofrequency (PRF) r modulates nerve activity through a weak pulsed electric current that preserves the structural integrity of nerve fibers, effectively alleviating pain and improving patient quality of life (11). PRF reduces the incidence of hyperalgesia, soreness, burning pain, and motor nerve injury, making it a preferred approach for neuropathic pain management, though it has been less frequently applied to GPN. Recent advancements in musculoskeletal ultrasound have enabled the visualization of peripheral nerve pulses during radiofrequency application (11–13). This study retrospectively evaluates the efficacy and safety of ultrasound-guided, long-duration high-voltage PRF in treating primary GPN. Unlike traditional radiofrequency ablation, high-voltage PRF modulates nerve signal conduction via pulse currents without destructively affecting nerve fibers, resulting in fewer complications such as numbness and motor dysfunction (14, 15). Extending PRF pulse duration enhances the suppression of pathological nerve activity while avoiding thermal damage to nerve structures (16). Evidence suggests that prolonged pulse durations contribute to reducing neural hyperactivity and facilitating the release of neurotrophic factors, such as brain-derived neurotrophic factor (BDNF) and glial cell line-derived neurotrophic factor (GDNF), which mitigate pain and reduce the risk of nerve injury (14, 17). Ultrasound enables real-time visualization of anatomical structures, including nerves and blood vessels, facilitating precise PRF needle placement and minimizing the risk of accidental punctures or damage to adjacent tissues, without exposing patients or practitioners to radiation. Consequently, ultrasound-guided PRF is being increasingly utilized in treating various types of neuralgia (18–20). Despite its growing application, systematic research on the specific use of ultrasound-guided PRF for primary GPN remains limited. This study aims to assess the efficacy and safety of high-voltage, long-duration PRF for GPN and to refine ultrasound-guidance techniques to further reduce post-procedural complications. These findings seek to substantiate future GPN treatments and broaden the applicability of PRF in managing other forms of neuralgia.

## 2 Materials and methods

### 2.1 Clinical data

This retrospective study received approval from the Clinical Trial Ethics Committee of the Affiliated Hospital of Southwest Medical University, which authorized the study protocol and granted a waiver for signed consent (registration number: KY2023186). The study adheres to the ethical standards outlined in the Declaration of Helsinki by the World Medical Association. The review encompassed patients diagnosed with glossopharyngeal neuralgia in the Pain Department of the Affiliated Hospital between August 2019 and October 2022. These patients, after unsuccessful drug treatment, received ultrasound-guided PRF therapy targeting the glossopharyngeal nerve for pain relief.

The inclusion criteria were as follows: (1) age above 18 years; (2) meeting all diagnostic criteria set by the International Headache Society (IHS) in ICHD-3 (21) (Table 1); (3) achieving over 50% reduction in Visual Analog Scale (VAS) scores and experiencing throat numbness following a diagnostic block (1 mL 2% lidocaine) prior to PRF therapy; (4) ineffectiveness of drugs or other treatments; and (5) patients who refuse microvascular decompression (MVD) or experience recurrence after MVD.

**Table 1:** Diagnostic criteria for glossopharyngeal neuralgia by the International Headache Association.

A. Recurring paroxysmal attacks of unilateral pain in the distribution of the glossopharyngeal nerve1 and fulfilling criterion B
B. Pain has all of the following characteristics:
1. Lasting from a few seconds to 2 minutes
2. Severe intensity
3. Electric shock-like, shooting, stabbing or sharp in quality
4. Precipitated by swallowing, coughing, talking or yawning
C. Not better accounted for by another ICHD-3 diagnosis.

The exclusion criteria were as follows: (1) patients with incomplete medical records; (2) patients with secondary glossopharyngeal neuralgia due to intracranial or extracranial lesions or trauma; (3) patients with a prior history of invasive treatments, such as intracranial microvascular decompression, extracranial glossopharyngeal vagotomy, or extracranial glossopharyngeal nerve radiofrequency ablation; (4) patients who did not undergo lingual-pharyngeal PRF during hospitalization; and (5) patients who received other treatments related to glossopharyngeal neuralgia after discharge.

### 2.2 Treatment procedures

**Figure 1:**
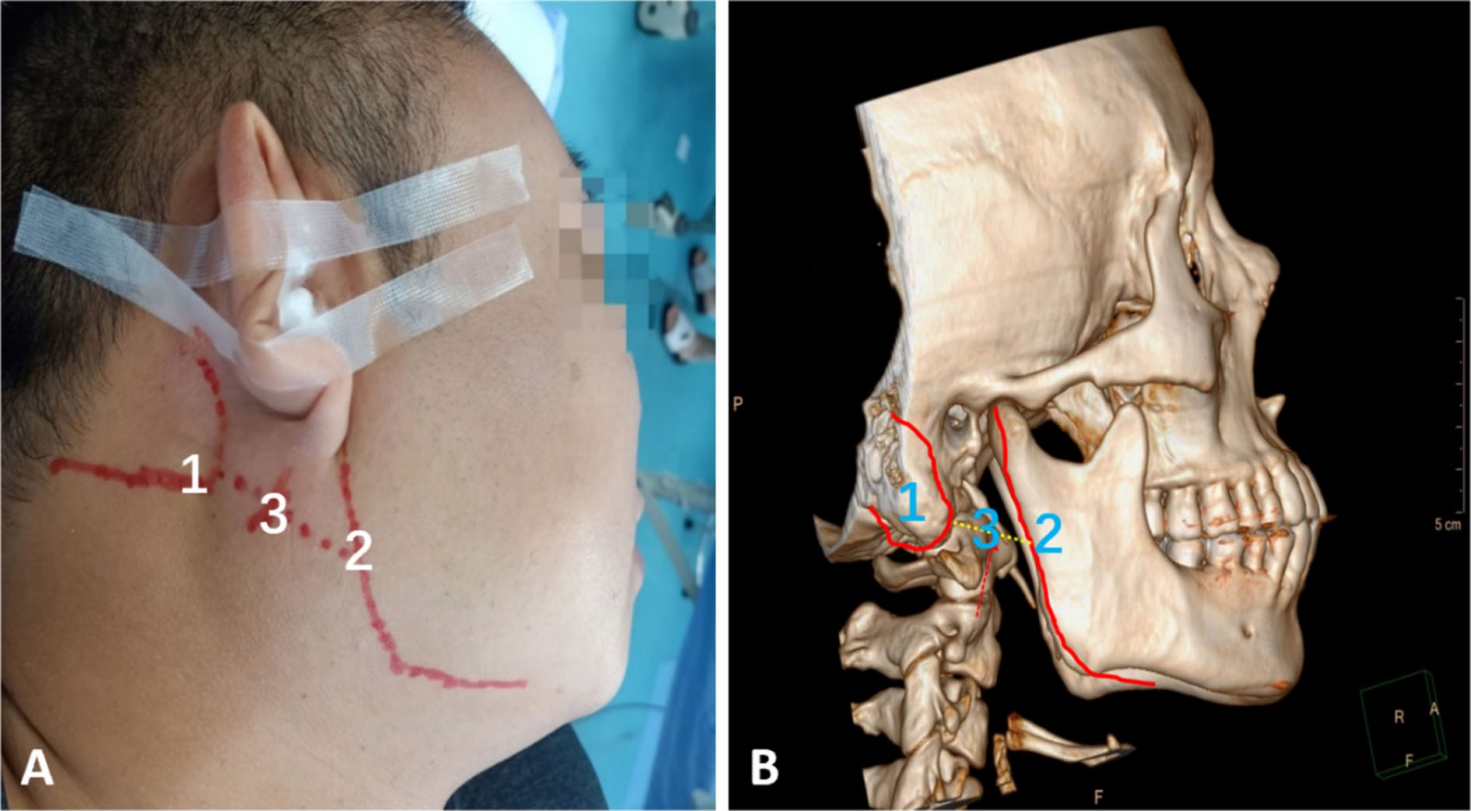
Patient’s body position and body surface markers **(A)** Body positioning: patient in a supine position with the head tilted to the opposite side. Label 1 indicates the mastoid, label 2 indicates the mandibular margin, and label 3 indicates the midpoint between the mastoid and mandibular margin. **(B)** CT 3-dimensional reconstruction of panel **(A)**.

**Figure 2:**
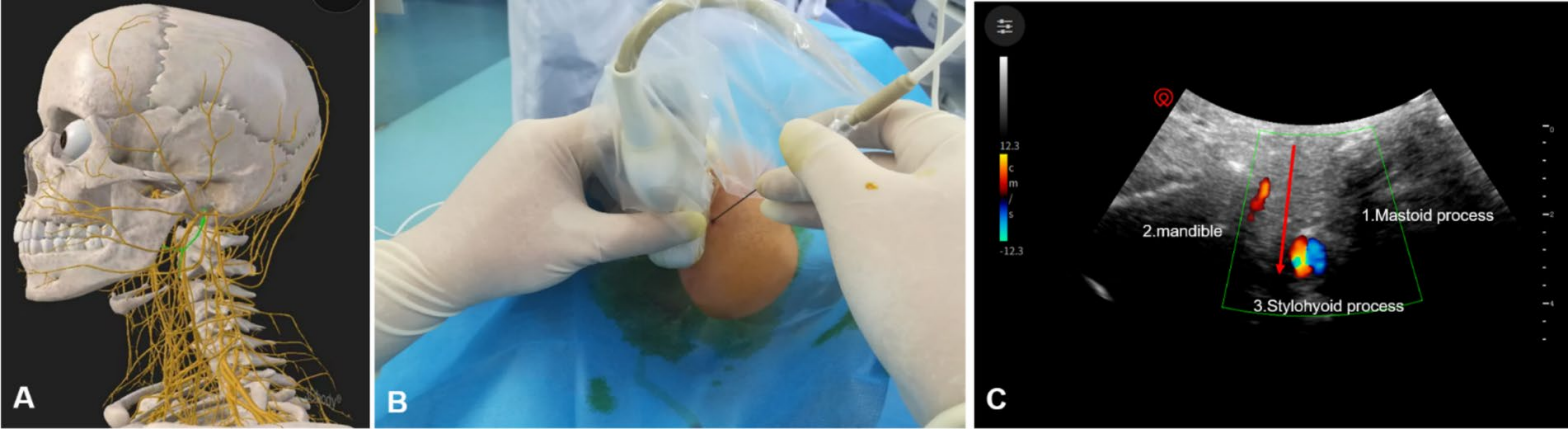
Ultrasound-guided glossopharyngeal nerve treatment. **(A)** Anatomical structure of the glossopharyngeal nerve. **(B)** Placement of the ultrasound probe and radiofrequency cannula. **(C)** Diagram of radio-frequency puncture needle reaching glossopharyngeal nerve under ultrasound guidance; red arrow, puncture path; 3, styloid process;1, mastoid process; 2, mandible.

All PRF treatments are performed by experienced pain physicians. Patients were positioned supine on the treatment bed, with their heads turned to the unaffected side to expose the treated side of the neck. A cotton ball was placed in the external auditory canal to prevent antiseptic solution from entering the ear canal, and the auricle was secured with tape. Under ultrasound guidance, the mastoid and mandible were marked with a red pen (Figure 1A). The puncture site was identified along the body surface between the midpoint of the mandibular margin and the midpoint of the mastoid process (Figure 1B). The treatment area was disinfected with iodophor and covered with disposable sterile drapes. Using a low-frequency convex array ultrasound probe with a sterile cover, 1% lidocaine was administered for local anesthesia. An external plane puncture technique was then performed, guided by ultrasound to position the radiofrequency cannula at the surface of the cavernous process. To avoid blood vessels, adjustments to the ultrasound plane were made to reach the end of the cavernous process (Figure 2). The stimulation parameter was set to 0.2–0.3 V at 50 Hz, effectively inducing sensations of pain, distension, or numbness in the glossopharyngeal nerve-innervated throat and tonsil areas, confirming precise localization. Pulsed radiofrequency therapy was then applied in 2 cycles at 72 V, 2 Hz, 20 ms, and 42°C for 6 min. At the conclusion of PRF, a 3 mL analgesic compound (lidocaine 40 mg, dexamethasone 5 mg, and 0.9% normal saline prepared to 10 mL) was injected adjacent to the glossopharyngeal nerve. Following the procedure, the patient’s vital signs were closely monitored for 10 min. Once stability and the absence of any discomfort or complications (e.g., nausea, vomiting, throat injury, hoarseness, choking, vasovagal reflex, or bleeding at the puncture site) were confirmed, trained staff safely returned the patient to the ward using a transport cart.

### 2.3 Efficacy evaluation

#### 2.3.1 Pain evaluation

Pain was assessed using the VAS score, recorded preoperatively and at 1 week, 1 month, 3 months, and 6 months postoperatively.

#### 2.3.2 Drugs use

Dosages of oxcarbazepine tablets and pregabalin capsules were recorded.

#### 2.3.3 Efficacy evaluation

Treatment efficacy based on pain relief was calculated using a VAS-weighted method, with categories defined as follows: excellent efficacy indicated complete pain resolution or > 75% VAS reduction; good efficacy corresponded to a 51–75% VAS reduction; effective was defined by a 26–50% VAS reduction; and poor efficacy was determined by a VAS reduction of ≤25%. The effective rate was calculated as follows: Effective rate = the number of cases with treatment efficacy defined as (excellent efficacy + good efficacy + effective) / total number of cases × 100 (%).

#### 2.3.4 Complications

Postoperative side effects, including neck swelling, hoarseness, dyspnea, dysphagia, pharyngeal numbness, petechiae, hematoma, and infection at the puncture site, were documented. The Barrow Neurological Institute-Numbness Scale (BNI-N) assessed cervicofacial and pharyngeal numbness severity (22), with the following grades: Grade I, no numbness; Grade II, mild numbness without significant impact on life; Grade III, pronounced numbness with minimal life impact; and Grade IV, severe numbness significantly affecting daily activities.

#### 2.3.5 Statistical analysis

Statistical analysis was conducted using IBM SPSS software (version 26, IBM Corporation, USA). Normally distributed continuous data were presented as mean ± standard deviation (SD), while non-normally distributed continuous data were reported as median and interquartile range (IQR). VAS scores and dosages of oxcarbazepine and pregabalin across time points were analyzed using one-way ANOVA, with Bonferroni adjustments for group comparisons. Discrete data were expressed as frequencies and percentages. A *p*-value of <0.05 was considered statistically significant.

## 3 Results

### 3.1 Baseline characteristic

Thirteen patients who received ultrasound-guided PRF treatment for glossopharyngeal neuralgia were included in this study. Following PRF, all patients reported hyperalgesia in the glossopharyngeal nerve-innervated areas. Each patient completed 6 months of follow-up, with 11 and 6 patients continuing through 1 and 2 years of follow-up, respectively. The mean follow-up duration was 20 ± 13.35 months, ranging from 6 to 48 months.

Table 2 details preoperative baseline and intraoperative characteristics, including patient demographics (age, sex, affected side, disease duration), comorbidities, and laboratory values (white blood cell count, lymphocyte count, albumin, globulin). Medication data for pregabalin and oxcarbazepine tablets are also provided. Of the 13 patients, 6 (46.15%) were male.

**Table 2:** Patient characteristics.

Patients	Total (*n* = 13)
Age (year)	59.61 ± 14.28
Gender, male, *n* (%)	6, 46.15%
Affected side, right, *n* (%)	8, 61.53%
Disease course (month)	27.94 ± 30.02
Leukocyte count (×10^9^/L)	6.04 ± 1.22
Lymphocyte count (×10^9^/L)	1.67 ± 0.67
Albumin (g/L)	43.8 ± 2.73
Immune globulin (g/L)	27.60 ± 4.28
Pregabalin capsule (mg)	80.76 ± 77.83
Oxcarbazepine tablet (mg)	292.3 ± 325.22
Tramadol hydrochloride capsules(mg)	30.8 ± 75.11
Preoperative VAS score	7.23 ± 0.92

Data in age are expressed as mean ± SD.

Generally, glossopharyngeal neuralgia presented as unilateral involvement, with right-side involvement observed in 8 patients (61.53%). All patients had either insufficient pain relief or recurrent symptoms prior to surgery, with a mean preoperative VAS score of 7.23 ± 0.92. Preoperatively, patients were taking one or more oral medications: seven patients (53.84%) were on pregabalin capsules (mean dose 80.76 ± 77.83 mg), 7 patients (53.84%) on oxcarbazepine tablets (mean dose 292.3 ± 325.22 mg), and 2 patients (15.38%) on tramadol sustained-release capsules (mean dose 30.8 ± 75.11 mg). Two patients on oral carbamazepine were excluded based on the study’s exclusion criteria.

### 3.2 Pain assessment

**Figure 3:**
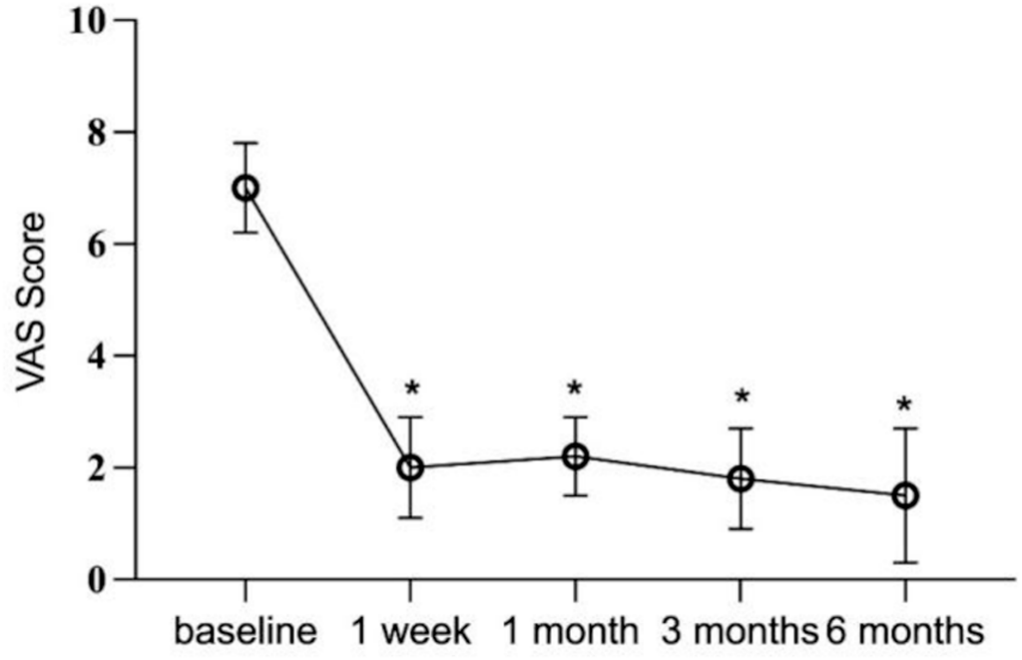
Postoperative VAS scores at each follow-up time point.

Immediately following PRF treatment, all patients recorded a VAS score of 0 due to the effects of local anesthetics. At the 1-week follow-up, 11 patients (84.61%) experienced a > 50% reduction in VAS scores. By the 6-month follow-up, 5 patients (38.46%) maintained a VAS score of 0, 6 (46.15%) reported mild pain, and 2 (15.38%) reported moderate pain. Across all 13 patients, postoperative VAS scores at each follow-up interval were significantly lower than preoperative scores (*p* < 0.05) (Figure 3). At 6 months postoperatively, 69.23% of patients demonstrated excellent and good efficacy, with an overall effectiveness rate of 84.61%. Poor efficacy was noted in 2 patients (15.38%) (Table 3).

**Table 3:** Evaluation of efficacy based on VAS score at 6-month follow-up (cases, %).

Category	Number of cases	Excellent efficacy	Good efficacy	Effective	Poor efficacy	effective rate
PRF	13	6 (46.15%)	3 (23.07%)	2 (15.38%)	2 (15.38%)	11 (84.61%)

### 3.3 Drugs use

**Figure 4:**
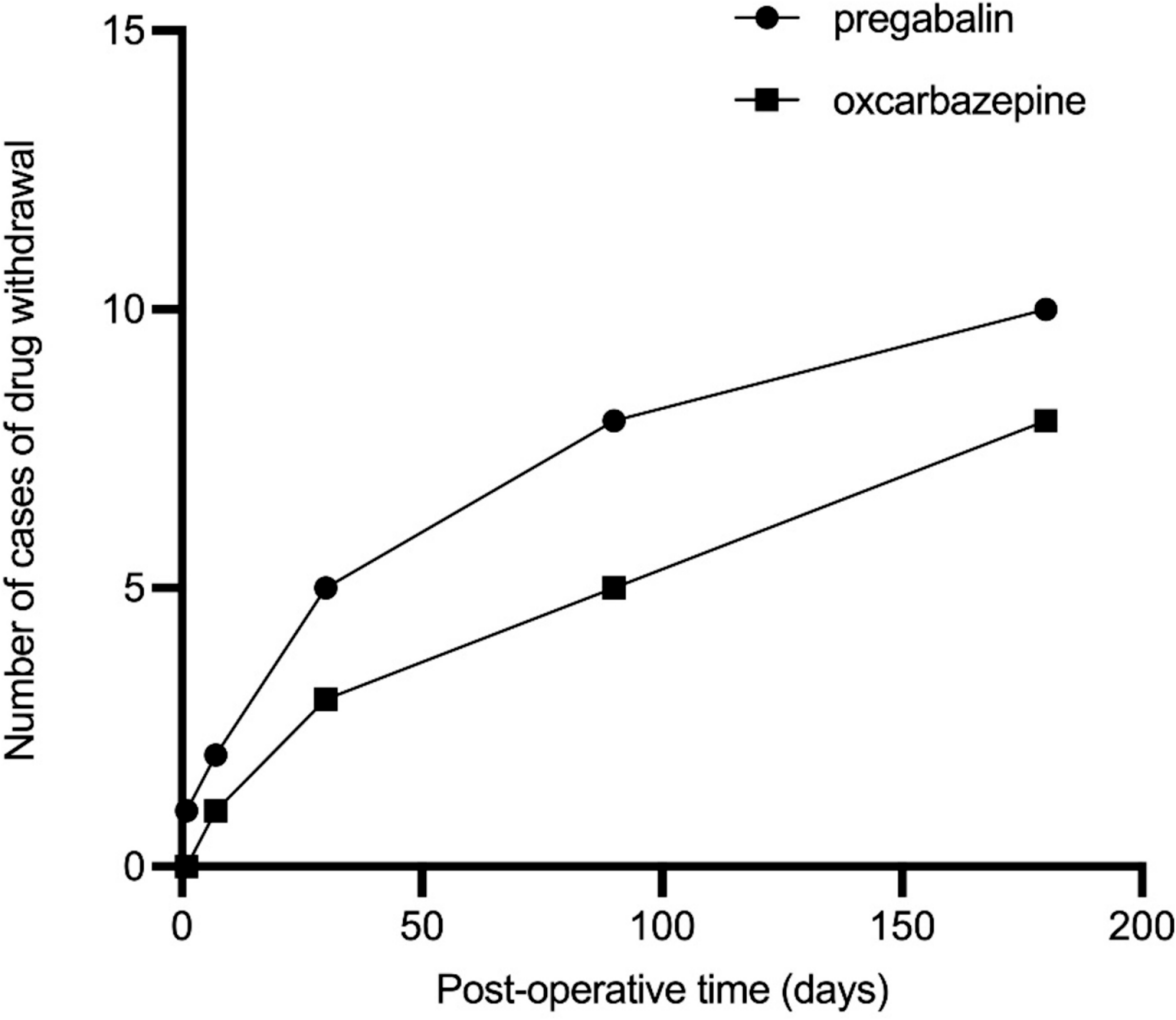
Number of cases of postoperative pregabalin and oxcarbazepine discontinuation (rate).

Postoperatively, the dosage of pregabalin capsules was significantly reduced in all 13 patients compared to preoperative levels (*p* < 0.05). The rates of pregabalin discontinuation were as follows: 1 patient (7.69%) on the day of PRF, 2 (15.38%) at 1 week, 5 (38.46%) at 1 month, 8 (61.53%) at 3 months, and 10 (76.92%) at 6 months postoperatively. For oxcarbazepine, the discontinuation rates were none (0.00%) on the day of PRF, 1 patient (7.69%) at 1 week, 3 (23.07%) at 1 month, 5 (38.46%) at 3 months, and 5 (61.53%) at 6 months postoperatively. Additionally, postoperative oxcarbazepine dosages were significantly lower in all patients compared to preoperative dosages (*p* < 0.05) (Figure 4).

### 3.4 Side effects

During PRF administration, patients maintained stable vital signs, with no major perioperative complications such as throat injury, hoarseness, choking, infection, or vagal-heart reflex. However, two patients (15.38%) experienced facial swelling, and one patient (7.69%) had minor bleeding after needle extraction, although no hematoma developed at the puncture site. Among the 13 patients, 84.61% (11) presented with a pharyngeal numbness score of grade I or II on the day of PRF treatment; by the 1-week follow-up, all patients had a numbness score of grade I. Numbness scores remained consistent at the 1-week, 1-month, 3-month, and 6-month follow-ups, with no significant changes in numbness observed at 1 and 2-year follow-ups compared to earlier postoperative assessments.

## 4 Discussion

GPN is characterized by severe paroxysmal cutting or electrical discharge-like pain localized to the glossopharyngeal nerve distribution, classifying it as neuropathic pain (9). It has a prevalence of approximately 0.7 per 10,000 individuals, predominantly affecting those over 35 years of age, with no observed gender differences. Pain typically presents unilaterally on the pharyngeal wall and the base of the tongue, occasionally affecting both sides, and may radiate to the mastoid process and deep ear regions. Common triggers include eating, talking, yawning, and coughing (9), significantly impacting daily activities. Minimally invasive interventions are considered when pharmacological treatment is ineffective or causes intolerable side effects. RF therapy has gained prominence in managing neuropathic pain due to its minimally invasive nature. However, RF targeting the glossopharyngeal nerve can result in adverse effects such as a choking sensation in the pharynx, taste loss, and xerostomia, as this nerve is involved in salivary secretion, taste perception in the posterior third of the tongue, and stylopharyngeus muscle movement, along with providing sensory input to the pharyngeal mucosa (23). These potential side effects limit RF’s applicability. Conversely, PRF offers a non-neurodestructive alternative with advantages such as minimal invasiveness, safety, and repeatability (24). Research by Li et al. (25) has shown that PRF, through low-frequency pulsed currents, disrupts nerve impulse conduction, reduces ectopic neural discharge, and produces additional effects, including inhibition of inflammatory factors, enhancement of endogenous analgesic factor release, and promotion of nerve cell repair by inducing secretion of BDNF and GDNF.

Bharti (8) treated 25 cases of GPN secondary to oropharyngeal cancer with PRF. Their findings indicated a 50% efficacy rate, with 92% of patients experiencing pain relief at the 3-month follow-up. Unlike our study, which focused on primary GPN, their cohort included only secondary GPN cases resulting from malignant tumors. Patients in Bharti’s study also received multimodal treatment, including cancer pain management with a 3-step drug regimen, tumor surgery, and radiotherapy. The PRF guidance techniques differed as well; their study utilized X-ray guidance, while ours employed ultrasound guidance. Our study demonstrated a substantial reduction in VAS scores post-PRF therapy for the glossopharyngeal nerve, from a preoperative score of 7.23 ± 0.92 to 1.61 ± 1.12 at 6 months postoperatively. At this follow-up, 84.61% of patients showed effective pain relief, with 69.23% achieving excellent efficacy. These outcomes highlight the benefits of PRF as a non-destructive modification of traditional radiofrequency therapy (26, 27), primarily leveraging pulsed current stimulation to avoid nerve damage. In our study, PRF parameters were set at 72 V, 42°C, for 2 cycles of 360 s each. Studies indicate that temperatures below 45°C do not cause significant nerve fiber damage while effectively treating neuropathic pain (28). Favorable results have also been observed by Tilburg et al. (11), who successfully used bilateral PRF in a patient with bilateral GPN, although with only a single patient and under X-ray guidance. Future studies with larger sample sizes are warranted to further evaluate the efficacy and safety of PRF for GPN treatment.

In this study, each patient received 720 s of PRF upon reaching the glossopharyngeal nerve, resulting in significant reductions in oxcarbazepine and pregabalin dosages post-treatment. These reductions highlight PRF’s effectiveness in managing primary GPN and improving patient quality of life. Discontinuation rates for pregabalin and oxcarbazepine were 76.92 and 61.53%, respectively, at 6 months post-treatment, suggesting that PRF not only effectively mitigates pain but may also serve as a viable alternative for patients dependent on long-term medication. This shift potentially improves quality of life by lessening reliance on neuropathic pain medications, such as gabapentin and oxcarbazepine, which, when used long-term, often cause side effects like fatigue, dizziness, and cognitive impairment, impacting patient well-being. Reducing dependence on these medications via PRF may also diminish the risks associated with drug tolerance, which is critical for chronic pain management. The safety and repeatability of PRF provide patients with more stable, long-term relief. As patients with GPN often exhibit variable responses to pharmacological treatments, PRF can fill the therapeutic gap for those intolerant to drugs or experiencing limited medication efficacy. These promising discontinuation rates indicate PRF’s potential value in comprehensive GPN management, allowing for a personalized approach suited to individual patient needs. Future studies should investigate PRF’s application across other types of neuralgia, with multi-center, large-sample prospective studies needed to confirm its long-term efficacy and safety.

In the immediate postoperative period, 84.61% of patients (11) presented with pharyngeal numbness scores of grades I to II, with all patients showing similar results at the 1-week follow-up. This finding suggests that PRF induces minimal sensory reduction in the glossopharyngeal nerve without causing postoperative nerve damage. By the 6-month follow-up, none of the patients experienced complications such as hematoma, infection, hoarseness, dry mouth, dysphagia, or dyspnea, underscoring the safety of the procedure. Bharti (8) reported transient facial paralysis in 2 patients with secondary GPN, but complications such as neck hematoma, dyspnea, or dysphagia were absent in our study, indicating that PRF may offer a safer profile for both primary and secondary GPN treatment. At 6 months post-treatment, PRF demonstrated an efficacy rate of 84.61% (11), with 2 patients (15.38%) showing poor outcomes. A study by Liu et al. (7) with 12 patients using varied treatments and evaluating outcomes based on a 2-point decrease in VAS scores reported an effective rate of 83.3% at 6 months, slightly lower than our study. Differences may be attributed to variations in inclusion criteria and treatment modalities, as Liu et al.’s study focused on nerve blocks, while our approach combined PRF with a nerve block (7). Case-by-case studies of radiofrequency for GPN have also shown similar outcomes. One patient in this study experienced recurrence and underwent a secondary treatment, achieving good efficacy, demonstrating PRF’s reproducibility and reliable therapeutic outcomes. Swain et al. (29) treated GPN resulting from an elongated styloid process with X-ray-guided PRF, but further case studies are necessary to validate PRF’s safety and efficacy across different patient profiles.

The target site for glossopharyngeal nerve PRF is situated deep within the caudate, closely surrounded by the internal carotid artery, internal jugular vein, spinal cord, pharyngeal cavity, and other critical structures. Real-time visualization is limited with CT or X-ray-guided puncture, which lacks sensitivity to surrounding blood vessels and nerves, thereby increasing the risk of inadvertent puncture-related complications. In contrast, this study employed ultrasound guidance, allowing dynamic, real-time observation of the needle’s trajectory, thereby reducing the likelihood of vascular puncture and nerve injury compared to CT and X-ray methods. Khan et al. demonstrated the advantages of ultrasound guidance for glossopharyngeal nerve blocks and magnetic therapy, while Teixeira et al. reported a positive correlation between field strength and PRF treatment efficacy. High-voltage, long-duration PRF is extensively applied in minimally invasive treatments for neuropathic pain, including trigeminal neuralgia and postherpetic neuralgia, providing enhanced therapeutic stability compared to conventional PRF techniques. However, few studies have focused on the optimal radiofrequency parameters specifically for GPN.

This study has certain limitations. Variability in patient factors, such as age, disease duration, and health status, may influence treatment outcomes, and changes in preoperative analgesic use may impact results. The study’s small sample size of 13 patients limits generalizability across the broader GPN population. Additionally, as a single-center retrospective study without a control group, the findings may be affected by the natural disease progression or placebo effects. Conducting a multi-center, prospective randomized controlled trial would be beneficial to substantiate the reliability of these outcomes.

## 5 Conclusion

In conclusion, ultrasound-guided, long-duration, high-voltage PRF has proven effective in alleviating GPN pain, improving quality of life, and providing reliable medium- and long-term outcomes with a reduced incidence of adverse effects. However, the optimal pulse duration and voltage parameters for PRF treatment of the glossopharyngeal nerve remain undetermined, highlighting the need for further investigation. This study’s retrospective design lacks the methodological rigor of a randomized, blinded trial, and the small sample size reflects the rarity of the condition. Future multicenter studies with larger cohorts are needed to validate long-term efficacy and establish standardized treatment protocols.

## Data Availability

The raw data supporting the conclusions of this article will be made available by the authors, without undue reservation.
