# Supplementary material for: The efficacy of ultrasound-guided pulsed radiofrequency in the treatment of primary glossopharyngeal neuralgia
Source: Front Neurol. 2024 Nov 28;15:1453598. doi: 10.3389/fneur.2024.1453598 (PMC11634690; doi:10.3389/fneur.2024.1453598)
Supplement: Supplementary file 1 [file Data_Sheet_1.PDF]

Supplementary Material

The information on the reduction in pregabalin dosage compared to the preoperative dose showed as supplementary material.

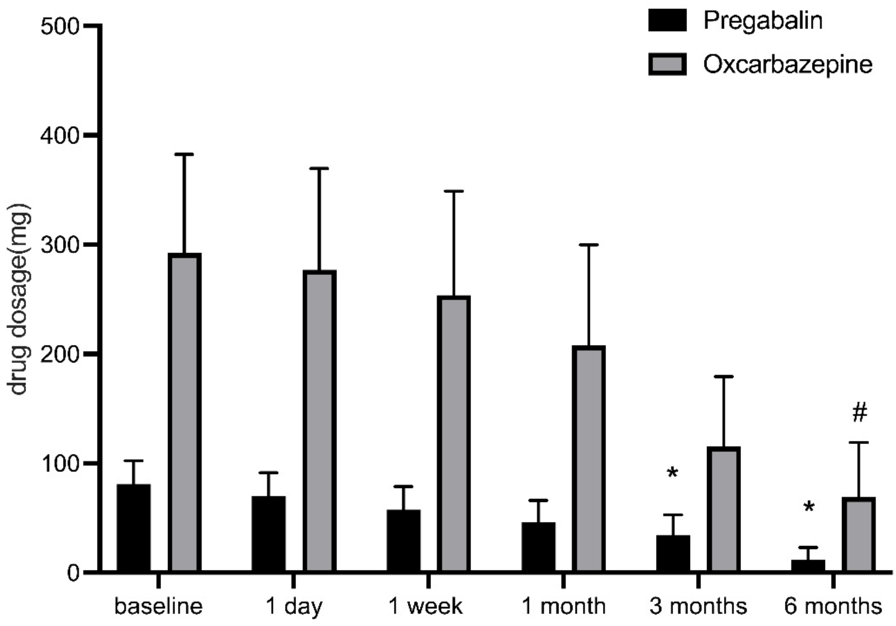

*compared with baseline in pregabalin
# compared with baseline oxcarbazepine
